# Clustering of Socioeconomic, Behavioural, and Neonatal Risk Factors for Infant Health in Pregnant Smokers

**DOI:** 10.1371/journal.pone.0008363

**Published:** 2009-12-18

**Authors:** Caren I. Lanting, Simone E. Buitendijk, Matty R. Crone, Dewi Segaar, Jack Bennebroek Gravenhorst, Jacobus P. van Wouwe

**Affiliations:** 1 Netherlands Organization of Applied Scientific Research (TNO) Prevention and Health, Leiden, The Netherlands; 2 STIVORO, Den Haag, The Netherlands; 3 Department of Public Health and Primary Care, Leiden University Medical Centre, Leiden, The Netherlands; The University of Adelaide, Australia

## Abstract

**Background:**

Tobacco smoking is a major cause of morbidity and mortality, including during pregnancy. Although effective ways of promoting smoking cessation during pregnancy exist, the impact of these interventions has not been studied at a national level. We estimated the prevalence of smoking throughout pregnancy in the Netherlands and quantified associations of maternal smoking throughout pregnancy with socioeconomic, behavioural, and neonatal risk factors for infant health and development.

**Methodology/Principal Findings:**

Data of five national surveys, containing records of 14,553 Dutch mothers and their offspring were analyzed. From 2001 to 2007, the overall rate of smoking throughout pregnancy fell by 42% (from 13.2% to 7.6%) mainly as a result of a decrease among highly educated women. In the lowest-educated group, the overall rate of smoking throughout pregnancy was six times as high as in the highest-educated group (18.7% versus 3.2%). Prenatal tobacco smoke exposure was associated with increased risk of extremely preterm (≤28 completed weeks) (OR 7.25; 95% CI 3.40 to 15.38) and small-for-gestational age (SGA) infants (OR 3.08; 95% CI 2.66 to 3.57). Smoking-attributable risk percents in the population (based on adjusted risk ratios) were estimated at 29% for extremely preterm births and at 17% for SGA outcomes. Infants of smokers were more likely to experience significant alcohol exposure in utero (OR 2.08; 95%CI 1.25 to 3.45) and formula feeding in early life (OR 1.91; 95% CI 1.69 to 2.16).

**Conclusions:**

The rates of maternal smoking throughout pregnancy decreased significantly in the Netherlands from 2001 to 2007. If pregnant women were to cease tobacco use completely, an estimated 29% of extremely preterm births and 17% of SGA infants may be avoided annually.

## Introduction

Tobacco smoking is a major cause of morbidity and mortality, including during pregnancy. Smoking in pregnancy is associated with very preterm birth (less than 32 weeks), fetal growth restriction and low birth weight [1], [2]. It increases the risk of sudden infant death syndrome, and it has been linked to an increased likelihood of asthma in childhood and a lower IQ in adulthood [3]–[7]. There are effective ways to help pregnant women to stop smoking and reduce preterm birth and low birth weight [8], but the impact of these interventions has not been studied at a national level in the Netherlands.

In most developed countries, there are marked social differences between women who smoke and those who do not, with continued smoking into pregnancy showing a strong association with socioeconomic disadvantage [9], [10]. Also, across the literature, smoking has been associated with low rates of breastfeeding [11]. In the present study, we report on the prevalence of smoking throughout pregnancy for 2001 to 2007 and quantify associations of maternal smoking throughout pregnancy with socioeconomic (low educational attainment, single parenthood), behavioural (alcohol consumption during pregnancy, initiation of formula-feeding), and neonatal (preterm birth and intra-uterine growth retardation) risk factors for infant health and development.

## Methods

The data of five national surveys, containing anonymous records of 14,553 Dutch mothers and their offspring were analyzed.

### Ethics Statement

The study design was approved by the Leiden University Medical Centre Medical Ethics Committee. The Committee did not require that informed consent was given for the surveys: return of the anonymous questionnaire was accepted as implied consent.

### Data Collection

Between 2001 and 2007, five nationwide surveys were carried out among mothers of infants under six months of age. Data were collected by means of a two-step procedure. Firstly, for each survey, each of the 65 organizations executing the Mother and Child Health Care program in the Netherlands were asked to provide names and addresses of five Well-Baby Clinics that were willing to participate. In our invitation to the organizations it was asked that they identify Well-Baby Clinics whose clients vary in socioeconomic status. Next, each participating Well-Baby Clinic randomly distributed anonymous questionnaires with stamped, pre-addressed return envelopes to the first 20 visiting mothers of infants under six months of age. Participants completed the questionnaires at home.

### Study Population

Per survey, 222 to 263 out of the 325 Well-Baby Clinics approached participated. From 2001 to 2007, a total number of 25,120 anonymous questionnaires were distributed, of which 15,428 (61.4%) were completed and returned. Of the completed questionnaires, 518 (0.03%) were excluded because the inclusion criterion regarding the age of the infant (≤6 months) was not fulfilled. A total number of 14,910 (59.4%) questionnaires were found eligible for analyses, 99.1% of which showed to be completed by the mother. Median age of the infant at completion of the questionnaire was two months (range zero to six months).

The sample was found to be representative of the Dutch female population with respect to maternal age at child birth, gestational age of the child, number of previous children, place of child birth (at home or in hospital), and residential region [12]. Differences did exist for level of formal education and immigrant status. In the standard Dutch population of 15- to 65-year-old women, 24% hold a degree on university or higher-professional level (2006) and 18% (2007) are born outside the Netherlands [13]. In our sample, these proportions were 36% and 6%, respectively.

### Questionnaire

#### Smoking

The questionnaire dealt with maternal smoking before, during, and after pregnancy, paternal or partner smoking, and daily number of cigarettes smoked.

#### Socioeconomic factors

Educational attainment of mothers was used as a measure of socioeconomic status. Formal education was categorized into low (primary or junior education), intermediate (secondary education), or high (higher-professional or university education) level. Mother's age (in years), whether she lived with a partner or not, and her country of birth (the Netherlands or elsewhere) were recorded.

#### Behavioural factors

Mothers recorded whether they initiated formula feeding or breastfeeding after the birth. In the 2007-survey, questions on alcohol consumption during pregnancy were added. Exposure during the first trimester, during the second or third trimester of pregnancy, and occasional abuse during pregnancy (more than six glasses per drinking occasion, also referred to as binge drinking) [14] were measured.

#### Neonatal factors

Mothers recorded parity (1/2/≥3), and gestational age (in completed weeks), birth weight (in grams), and gender of the infant. Gestational age was categorized into extremely preterm (≤28 completed weeks), very preterm (28 to 31 weeks), moderately preterm (32 to 36 weeks), and term (≥37 weeks) birth. In order to assess fetal growth, we used Dutch references curves [15]. Infants whose birth weight was below the 10th percentile at the corresponding gestational age, adjusted for gender and parity, were classified as small for gestational age (SGA).

### Data Analyses

Frequency tables and 95%-confidence intervals (95%CIs) were calculated for smokers and non-smokers. Differences between groups were assessed by means of Student's t, Mann-Whitney or chi-square tests, wherever appropriate. Mantel-Haenszel linear-by-linear association tests were used to identify trends. Relationships with socioeconomic, behavioural and neonatal factors were studied by means of (multinomial) logistic regression techniques. Because effects of stopping smoking at different stages during pregnancy differ [16], women who smoked throughout pregnancy (smokers) were compared to women who did not smoke at all during pregnancy (non-smokers). During the analyses, adjustments were made for: maternal educational attainment, age, single motherhood, parity, maternal country of birth, and child's gender. Strengths of associations are presented as crude and adjusted odds ratios (ORs) and 95% confidence intervals (95%CIs). Smoking-attributable risk percents among smokers and for the population (smokers and non-smokers) were calculated based on adjusted ORs, according to the method by Cole and MacMahon [17]. Population attributable risk percents may be used to judge priorities in public health, as they quantify the potential impact of risk-factor intervention programs [18]. Presented p-values are two-sided; p-values ≤0.05 were classified as statistically significant. Statistical analyses were performed with SPSS 14.0.

## Results

### Prevalence and Trends

Of 14,553 participating mothers, 9.7% (n = 1416) reported to have smoked throughout pregnancy. Median cigarette consumption was five per day (range 1 to 60). Of the participating mothers, 25.6% (n = 3166) indicated to have smoked during the six months prior to pregnancy. During the first six months after the birth, 14.0% (n = 2074) of mothers reported to smoke. Both before and after pregnancy, the median number of cigarettes smoked by the mother was 10 per day (range 1 to 60). Of fathers, 27.4% (n = 4025) smoked, with a median daily cigarette consumption equal to 10 (range 1 to 83). 31.6% (n = 4635) of all infants lived together with at least one smoker (mother and/or father). In smoking households, median consumption of cigarettes amounted to 24 per day (range 2 to 119).

**Figure 1:**
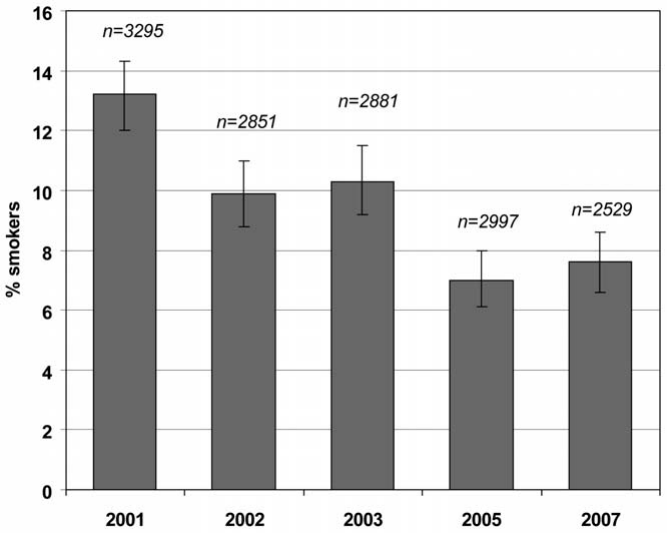
Prevalence of smoking throughout pregnancy in the Netherlands 2001–2007.

**Figure 2:**
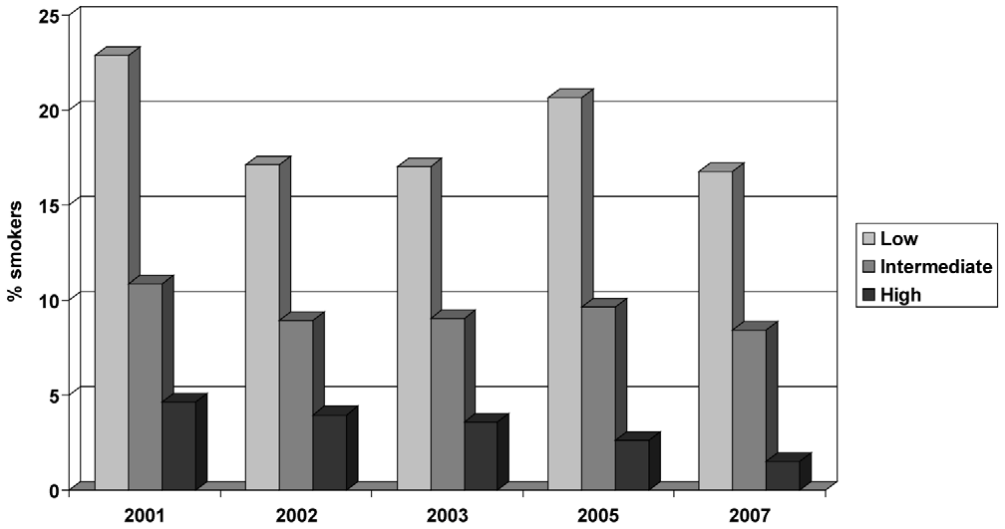
Prevalence of smoking throughout pregnancy by level of formal education (low, intermediate or high) in the Netherlands 2001–2007.

Between 2001 and 2007, the overall rate of smoking throughout pregnancy declined from 13.2% to 7.6% (42% reduction; p<0.001; fig. 1). Median cigarette consumption during pregnancy did not change over time. Sharp decreases in rates of smoking throughout pregnancy were seen between the surveys of 2001 and 2002 (25% reduction), and between 2003 and 2005 (32% reduction) (both p-values <0.001). In the highest-educated group, a statistically significant decrease in smoking rates over the years was observed (p<0.001); the proportion of smokers fell from 4.7% to 1.6% (66% reduction; fig. 2). The overall rate was 3.2%. Among intermediate-educated women, the proportion of smokers decreased from 12.6% to 8.5% (33% reduction), with an overall rate of 9.5%. This decrease did not reach significance (p = 0.06). In the lower-educated group, there was no statistically significant change over time. The overall rate of smoking throughout pregnancy for mothers with less education was 18.7%.

### Socioeconomic Factors

Pregnant smokers tended to belong to the lower-educated group (OR 6.31; 95% CI 5.31 to 7.50) and to be single mothers (OR 2.41; 95% CI 1.75 to 3.32) (table 1). As compared to non-smokers, smokers more often were born in the Netherlands (OR 1.62; 95% CI 1.23 to 2.15). They also tended to be somewhat younger at child birth (OR 1.02; 95% CI 1.01 to 1.04). Mean age at birth was 30 years for smokers versus 31 for non-smokers (table 1). And, if they had a partner, smokers were more likely to live with a partner who also was a current smoker (OR 7.81; 95% CI 6.90 to 8.92).

**Table 1:** Logistic regression analysis of several maternal characteristics on smoking status in pregnant women in the Netherlands 2001–2007.

Variable		Smokers	Non-smokers		Crude OR (95% CI)	Adjusted OR (95% CI)*
Age	n	1403	13068			
	Mean (sd; years)	30 (4.92)	31 (4.21)		1.06 (1.05–1.08)	1.02 (1.01–1.04)
Single mother	n	1405	13093			
	Yes/no (%)	4/96	2/98		3.11 (2.31–4.15)	2.41 (1.75–3.32)
Socioeconomic status	n	1366	12578	L/I	2.23 (1.97–2.52)	2.24 (1.97–2.54)
	L/I/H# (%)	45/41/14	20/40/40	L/H	6.60 (5.57–7.82)	6.31 (5.31–7.50)
Country of birth	n	1410	13079			
	Netherlands/other	95/5	94/6		1.35 (1.05–1.74)	1.62 (1.23–2.15)
Smoking status partner	n	1373	12995			
	Smoker/non-smoker	71/29	22/78		8.70 (7.69–9.90)	7.81 (6.90–8.92)
Occasional alcohol abuse while pregnant	n	140	1699			
	Yes/no (%)	18/82	9/91		2.27 (1.43–3.57)	2.08 (1.25–3.45)
Formula feeding	n	1412	13108			
	FF/BF$ (%)	38/62	19/81		2.60 (2.32–2.92)	1.91 (1.69–2.16)

OR. Odds ratio.

CI. Confidence interval.

# L = low; I =  intermediate; H = high.

* Odds ratios are mutually adjusted for: maternal age, single motherhood, parity, maternal level of formal education, maternal country of birth.

$ FF = formula feeding; BF = breast feeding.

### Behavioural Factors

#### Alcohol consumption

Overall, 28.6% (n = 526) of mothers reported social drinking during the first trimester of pregnancy. Of drinking mothers, 43.7% (n = 222) had less than one glass per month, 28.8% (n = 146) drank one to three glasses per month, and 27.5% (n = 140) drank more than three glasses per month. With respect to the second and third trimester of pregnancy, 24.3% (n = 497) of mothers reported social drinking: 62.8% (n = 309) had less than one glass per month, 22.4% (n = 110) drank one to three glasses per month, and 14.8% (n = 73) drank more than three glasses per month. Proportions of social drinking did not differ for smokers and non-smokers. 9.4% (n = 173) of participants reported occasional alcohol abuse during pregnancy (more than six glasses per drinking occasion). For smokers, this was 17.9% versus 8.7% for non-smokers (OR 2.08; 95%CI 1.25 to 3.45; table 1).

#### Formula feeding

In our study group, 21.1% (n = 3132) of mothers initiated formula feeding. 38.2% of smokers opted for formula feeding versus 19.2% of non-smokers. The difference remained after adjustment for maternal socioeconomic factors (OR 1.91; 95% CI 1.69 to 2.16) (table 1).

### Neonatal Factors

#### Pregnancy duration

Of all infants 5.6% were born preterm: 0.2% extremely preterm (≤28 weeks), 0.5% very preterm (28 to 31 weeks), and 4.8% moderately preterm (32 to 36 weeks). Median gestational age was 40 weeks for infants of smokers as well as for infants of non-smokers (5^th^ percentile 35 weeks, 95^th^ percentile 42 weeks, for both groups). After adjustments for maternal characteristics, the odds for a smoking mother, as compared to a non-smoking mother, to have an extremely preterm baby (≤28 completed weeks) were 7.25 (95% CI 3.40 to 15.38) (table 2). Proportions of very preterm or moderately preterm deliveries were equally divided among smokers and non-smokers. Smoking-attributable risk percents were 0.81 among smokers and 0.29 in the population (smokers and non-smokers).

**Table 2:** (Multinomial) logistic regression analysis of maternal smoking status throughout pregnancy on preterm birth and intrauterine growth in the Netherlands 2001–2007.

Variable	Category		Smokers	Non-smokers	Crude OR (95% CI)	Adjusted OR (95% CI)*
Gestational age at birth$		N	1416	13039		
	Extremely preterm	n	18	15	11.24 (5.65–22.36)	7.25 (3.40–15.38)
		%	1.3	0.1		
	Very preterm	n	10	63	1.49 (0.76–2.91)	1.23 (0.71–2.79)
		%	0.7	0.5		
	Moderately preterm	n	71	622	1.07 (0.83–1.38)	1.05 (0.83–1.22)
		%	5.0	4.8		
	Term	n	1317	12339	1.00	1.00
		%	93.0	94.6		
Birth weight for gestational age		N	1381	12924		
	<10^th^ percentile	n	324	1141	3.17 (2.76–3.64)	3.08 (2.66–3.57)
		%	23.5	8.8		
	≥10^th^ percentile	n	1057	11783	1.00	1.00
		%	76.5	91.2		

OR. Odds ratio.

CI. Confidence interval.

* Odds ratios are adjusted for maternal social factors (age, single motherhood, level of formal education country of birth). Results on gestational age are also adjusted for parity and gender of the newborn.

$ Extremely preterm: ≤28 completed weeks; very preterm: 28–31; moderately preterm: 32–36 weeks; term ≥37.

#### Intrauterine growth

Overall, 10.2% of all infants had birth weights below the 10^th^ percentile for their respective gestational age. Among smokers, 23.5% of infants were SGA (birth weight <10^th^ percentile for gestational age). Among non-smokers this was 8.8% (OR 3.08; 95% CI 2.66 to 3.57). Mean (± standard deviation) birth weight of infants born to smokers was 3240 g±566 g versus 3516 g±571 g for infants born to non-smokers. The smoking-attributable risk percents were 0.67 and 0.17, respectively among smokers and in the general population.

## Discussion

The overall rate of smoking throughout pregnancy fell by 42% from 2001 to 2007 in the Netherlands. This downward trend was mainly seen in the highest-educated group, and could not be detected in the lower-educated groups. Overall, in the Netherlands, 10% of women smoked throughout pregnancy.

At a European level, the percentages of smokers during pregnancy vary from 5% to 7% in Lithuania, the Czech Republic, Sweden, and Malta to 16% in Denmark and 21% in France [19]. Studies on alcohol and cigarette consumption are prone to under-reporting. This might lead to underestimation of prevalences.

During recent decades, rates of smoking in pregnancy declined, in response to a general policy of discouraging smoking. In 1982, in the Netherlands, 46% of women smoked during pregnancy [20]. In 1996, the proportion of smokers had fallen to 21% [21]. We observed sharp decreases in 2001, and from 2003 to 2005. The 2003–2005 decrease coincided with the implementation of the brief intervention strategy V-MIS in the Netherlands. V-MIS is a 5 A's based smoking cessation protocol (Ask, Advise, Assess, Assist, Arrange) which has been proven to be effective in lowering smoking prevalence among pregnant women [22], [23].

From the present study it becomes clear that maternal smoking in pregnancy is not just a health risk in and of itself, but a proxy indicator for several other health risks as well. As compared to non-smokers, pregnant smokers are six times more likely to be of lower socio-economic status. By contrast, smokers are less likely to be immigrants, which probably reflect cultural differences in smoking habits in women. Throughout the literature, low socioeconomic status has been associated with poorer health and more frequent unhealthy behaviours such as smoking. The mechanism behind this association is complex and remains to be disentangled, but there are indications that psychological stress has a role. Compared with the more advantaged, individuals of low socio-economic status are believed to experience greater and more severe daily stressors. Smokers report that smoking helps them to deal with stress and anxiety [24]. Pregnant women are vulnerable to pressures and report feeling constantly judged by others. [24]. Also, findings show that significant psychological symptoms and strains are being reported amongst pregnant women who smoke [25], [26]. Health risk perceptions may have a mediating role in the association between socio-economic status and smoking [27].

Occasional alcohol abuse during pregnancy was reported twice as much by smokers, as compared to non-smokers. This may well be related to elevated stress levels in the smoking pregnant population the more since proportions of social drinking did not differ for smokers and non-smokers. The highest levels of exposure occurred during the first trimester. Well known effects of fetal exposure to high levels of alcohol include facial anomalies, behavioural and CNS abnormalities, and growth restriction [14]. Prenatal exposure to alcohol as well as to cigarette smoke increases the risk of preterm birth and low birth weight. Based on our data it can be estimated that in the Netherlands a yearly number of 3000 unborn babies are being exposed to the combined risks of smoking and (occasional) alcohol abuse during pregnancy.

In addition, pregnant women who smoked were twice as likely to choose formula feeding over breastfeeding. Breastfeeding is the preferred method of feeding to achieve optimal child health, growth, and development [28]. Since both smoking and formula feeding are more common among the lower-educated, adjustments were in our analysis for educational differences between groups. Smokers not only initiate formula feeding more often, if they start breastfeeding, they tend to terminate early [29], [12]. In the literature, several possible explanations for the adverse relation between cigarette smoking and breastfeeding have been offered [30]. On the one hand, plausible physiological explanations exist. It has been suggested that nicotine in the maternal blood stream acts on human milk production by reducing the level of prolactin and that nicotine in human milk increases infant irritability and, hence, feeding difficulties [30]. Psychosocial explanations involve smokers being more insecure about their ability to breastfeed and less health conscious in general [30]. Until now the exact underlying mechanism remains unclear.

Almost all children of mothers who smoke during pregnancy will be exposed to environmental tobacco smoke in the postnatal period. And, even if smoking occurs outside the home and away from the infant, this does not seem to fully protect a smoker's infant from environmental tobacco smoke exposure due to contamination of dust and surfaces in homes of smokers. Environmental tobacco smoke exposure, as measured by cotinine levels in infant urine, was five to seven times higher in households of smokers trying to protect their infants by smoking outdoors, than in households of non-smokers [31]. Between 2001 and 2007, 32% of all Dutch infants were exposed to at least one smoker (mother and/or father), with a median level of exposure to 24 cigarettes per household per day. In 1996, 44% of the Dutch six-months-olds were exposed to one or more smokers in the family although the level of exposure was lower: on average between five and 15 cigarettes per day [20].

Women who smoked were twice as likely to be single mothers. In addition, women with a partner who also was a current smoker were eight times as likely to smoke during pregnancy, as compared to women with a partner who did not smoke. In a Cochrane review by Park and co-workers (2004) it is concluded that there is a strong association between partner support and successful smoking cessation [32]. In particular cooperative behaviours, such as talking the smoker out of smoking a cigarette, and reinforcement, were found to predict successful quitting. Negative behaviours, such as nagging and complaining, were found predictive of a relapse. Still, intervention programs that intend to improve partner support do not seem to increase long-term quitting rates, presumably because the interventions have not successfully changed the support provided [32].

We estimated that in women who, on average, smoke a daily number of five cigarettes throughout pregnancy, 81% of the total risk of extremely preterm birth and 67% of the total risk of SGA outcome can be attributed to tobacco smoke exposure. Other factors, such as socioeconomic status, maternal age, and parity, account for the remainder. We also estimated that at a population level (smokers and non-smokers), 29% of extremely preterm births and 17% of SGA outcomes can be prevented if pregnant women would refrain from smoking. In absolute terms this implies that, if pregnant smokers were to stop tobacco use completely, 12 extremely preterm and 170 SGA outcomes per 10,000 live births would be prevented annually in the Netherlands (181,336 live births per year [13], of which 0.4% extremely preterm (2004) [19]). Smoking-attributable risk percents were based on adjusted risk ratios. Factors we did not measure include psychosocial stress, multiple pregnancy, eating habits, and the level of health care during pregnancy. These may have caused residual confounding. The true preventive effect of smoking cessation may therefore be smaller.

In practice this gain will likely not be met soon without a much stronger concerted effort. Pooled data from 65 trials on effects of behavioural interventions for stopping smoking in pregnancy showed an absolute reduction of 6% in continued smoking [8]. A comparable reduction was observed in the Netherlands between 2001 and 2007. Such an effort would be extremely worthwhile even just in monetary terms. Based on our data, the 6%-smoking reduction has lead to an estimated annual prevention of six extremely preterm and 93 SGA outcomes per 10,000 live births. The incremental hospitalization costs roughly are €50,000 for extremely preterm and €5,000 for SGA infants [33]. Clearly, a stronger, more effective promotion of smoking cessation during pregnancy can have a significant effect on maternal and infant health, as well as on health care expenditure.

In conclusion, although the rates of maternal smoking throughout pregnancy decreased significantly in the Netherlands, one out of ten women still smoke throughout pregnancy. Children of smokers are more likely to face the consequences of preterm birth and fetal growth restriction. In addition, this group tends to be born into social disadvantage, is more likely to suffer significant alcohol exposure in utero, and is formula fed more often in early life. If pregnant women were to cease tobacco use completely, an estimated 29% of extremely preterm births and 17% of SGA infants may be avoided annually and at least 26 million euro would be saved on health care costs annually in the Netherlands alone.
